# Clinical and Biological Insights into Myelodysplastic Neoplasms Associated with Deletions of Chromosome 5q Region

**DOI:** 10.3390/hematolrep17060067

**Published:** 2025-11-29

**Authors:** Ugo Testa, Germana Castelli, Elvira Pelosi

**Affiliations:** Department of Oncology, Istituto Superiore di Sanità, Viale Regina Elena 299, 00161 Rome, Italy

**Keywords:** leukemia, myelodysplasia, chromosome abnormalities, karyotype, mutational profile, genome analysis, molecular classification

## Abstract

The only cytogenetic alteration defining a subtype of a myelodysplastic syndrome is represented by the deletion of the long arm of chromosome 5 (del(5q)), now classified as MDS with isolated del(5q). This subtype is associated with a peculiar phenotype mainly dependent on the haploinsufficiency of several genes located on the deleted arm of chromosome 5. These patients show a good prognosis and respond to treatment with lenalidomide, but some cases progress to acute myeloid leukemia. Molecular studies have, in part, elucidated the heterogeneity of MDS with isolated del(5q), mainly related to the association with different co-mutations that may affect leukemic transformation and survival. In other MDS patients, del(5q) is combined with other chromosomal abnormalities, giving rise to a condition of complex karyotype, associated with frequent *TP53* mutations and with a poor prognosis. Two different molecular pathways seem to be responsible for the generation of MDS with isolated del(5q) or of MDS with del(5q) associated with a complex karyotype.

## 1. Introduction

The discovery of 5q deletion associated with MDS was related to the characterization of three patients displaying a similar chromosomal abnormality in bone marrow and a similar hematological phenotype: the chromosomal abnormality consisted in a deletion at the level of the long arm of one chromosome 5; the hematological phenotype consisted of a macrocytic anemia refractory to conventional treatments, associated with normal or increased platelet counts and normal or decreased white blood cell count [1].

After this initial report, this hematological disorder associated with a 5q chromosomal abnormality was observed in other patients and was defined as 5q^−^ syndrome. In the 2001 World Health Organization (WHO) classification of myeloid neoplasms, this disorder was classified as a subtype of mDS and was termed MDS with isolated deletion 5q [2].

The diagnostic criteria for MDS associated with 5q deletion have been subsequently updated in the more recent classifications of myeloid neoplasms [3,4]. A 5q deletion is observed in MDS, not only as an isolated abnormality, but also in the context of other chromosomal abnormalities (complex karyotype and CK) and frequently in association with *TP53* mutations. This review analyzes the recent developments in the understanding of the pathogenesis and the prognostic impact of the different MDS subtypes bearing del(5q).

## 2. 5q Deletion in MDS

A 5q deletion represents one of the most frequent chromosomal abnormalities observed in MDS. MDS with 5q deletion can be subdivided into two groups: a group in which 5q deletion is either the sole cytogenetic abnormality or with one additional cytogenetic abnormality excluding −7/del(7q), and a group in which del(5q) is associated with other cytogenetic abnormalities in the context of a complex karyotype (CK). The distinction of these two groups of MDS with del(5q) is fundamental both at the biological and clinical levels. These two groups of MDS correspond to 2 of the 16 molecular subgroups identified for their unique molecular profiles, clinical phenotypes and disease courses [5].

The del(5q) group of MDS with isolated 5q abnormality is usually characterized at the level of hematological phenotype by low bone marrow blast cell counts and lowered Hb levels (lower than in the rest of MDSs) and with increased or normal platelet levels (higher than in the rest of MDS); these MDSs are predominantly observed in females (75% of cases) and are associated with a favorable OS compared to the rest of MDS [5]. A total of 84% of these patients had at least one gene mutation, the most frequent being *SF3B1* (22%), *DNMT3A* (21%), *TET2* (17%), *ASXL1* (14%) and monoallelic *TP53* mutations (13%) [5]. Interestingly, the mutations of four genes, *CSNK1A1* (10%), *IRF1* (5.4%), *RAD50* (4.1%) and *NFE2* (3.6%), located on 5q, are enriched in del(5q) MDS [5]. Importantly, the study of variant allele frequency (VAF) of the additional mutations *SF3B1, DNMT3A, TET2* and *ASXl1* suggests that these are events secondary to del(5q) [5].

Meggendorfer et al. explored a group of 123 MDS patients with isolated del(5q) for the presence of mutations using a panel of 27 genes frequently mutated in MDS and showed that about 40% of patients had no mutations and about 50% one mutation [6]. The genes most frequently mutated were *SF3B1* (19%), *DNMT3A* (18%), *TP53* (18%), *TET2* (12%), *CSNK1A1* (12%), *ASXL1* (6%) and *JAK2* (6%) [6]. The comparison of the mutation profile of various MDS subtypes showed that the mutation profile of MDS-del(5q) and MDS non-del(5q), *TP53* mutations were more frequent in MDS with isolated del(5q) compared to the rest of MDS (18% vs. 6%, respectively) [6]. In these patients, the presence of *SF3B1* mutations correlated with the presence of ring sideroblasts, while *JAK2* mutations correlated with elevated platelet counts [6]. At the level of the effect of mutations on prognosis, the presence of *SF3B1* mutations was associated with shorter survival compared to patients without *SF3B1*; however, the survival of *SF3B1*-mutant MDS with isolated del(5q) was similar to that observed in *SF3B1*-mutant MDS without del(5q) [6].

**Figure 1:**
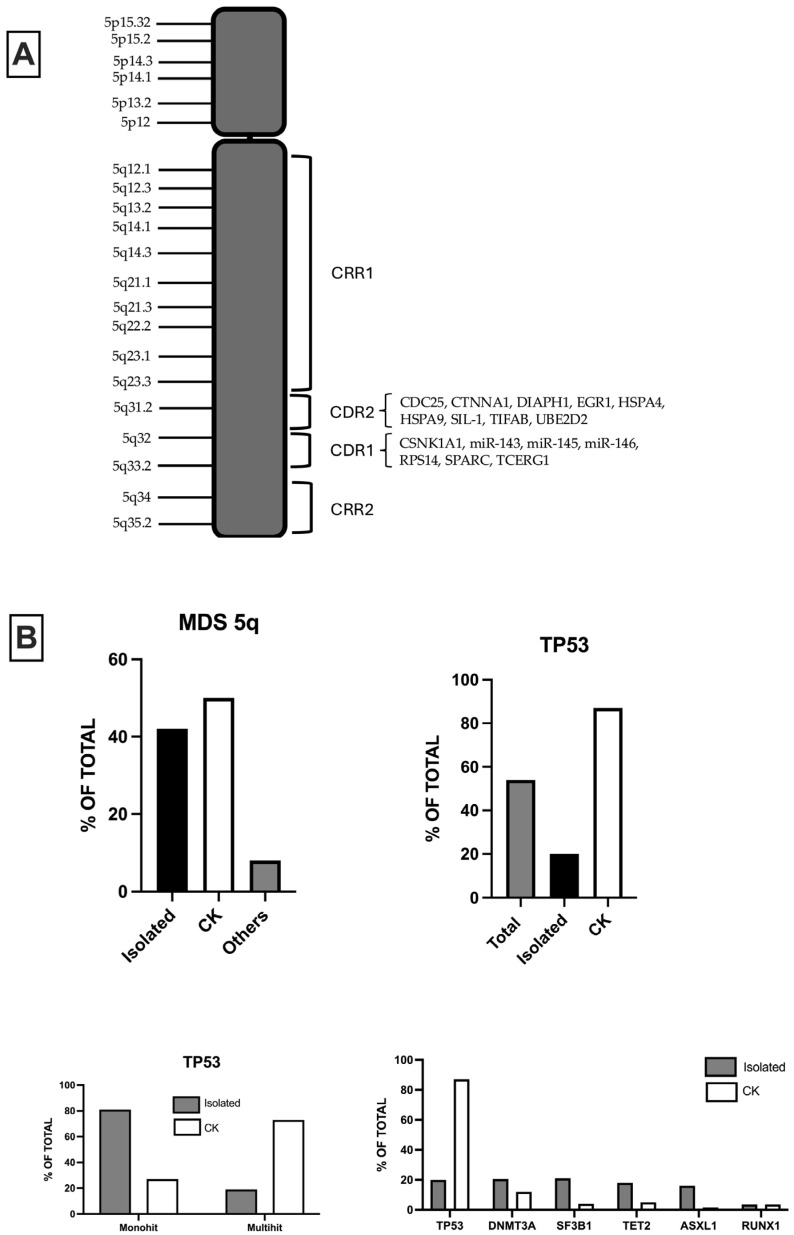
(**A**) Schematic representation of human chromosome 5. The CRRs and CDRs present in the 5q arm are outlined. The main functionally relevant genes located at the level of proximal and distal, CDRs are listed. (**B**) Cytogenetic and mutational profile of MDS-del(5q). Top left panel: Frequency of MDS-del(5q) as an isolated del(5q) abnormality or in the context of a complex karyotype or in association with another chromosome abnormality (others). Top right panel: frequency of *TP53* mutations in MDS-del(5q), analyzed as total, with isolated del(5q) or del(5q) in the context of CK. Middle panel: frequency of mono-hit and multi-hit *TP53* alterations among MDS with isolated del(5q) or in the context of CK. Bottom panel: most recurrent mutations in MDS with isolated del(5q) or in the context of CK.

A part of MDS patients with del(5q) display *TP53* mutations (Figure 1). Montoro et al. analyzed a group of 628 patients with isolated del(5q); 18.9% of these patients displayed *TP53* mutations: 24% of these patients with multi-hit *TP53* alterations and 76% with mono-hit alterations [7]. Although the mOS was similar in *TP53*-WT and *TP53*-mut 5q-deleted MDS, *TP53* multi-hit alterations and *TP53* mono-hit alterations with VAF > 20% were predictive of an increased risk of leukemia transformation [7]. Thus, the impact of VSF on monoallelic *TP53* alterations was evident: VAF <20% displayed a behavior similar to *TP53*-WT, and those with VAF >20% showed a behavior like multi-hit *TP53* alterations [6]. Finally, MDS del(5q) with monoallelic *TP53* alterations with VAF >20% displayed a significantly reduced mOS compared to MDS del(5q) *TP53*-WT [7]. The co-mutation profile of MDS del(5q) *TP53*-WT and *TP53*-mutated was similar [7].

Xie et al. evaluated the clinical correlation and the prognostic impact of cytogenetic clone size in MDS patients; their study included a cohort of 1001 MDS patients, comprising also 144 patients with isolated del(5q) [8]. In these patients, clone size exhibited a significant negative correlation with hemoglobin levels and a positive correlation between clone size and platelet levels [8]. Among the various MDS cytogenetic groups, those with isolated MDS del(5q) had the longest mOS (65.9 months) and mLFS (58.4 months) [8]. The observation that clonal sizes are associated with increased anemia and thrombocytosis supports the view that clonal expansion contributes to the development of these disease manifestations [8].

The other group of del(5q) MDS is associated with TP53-complex MDS and CK MDS; the TP53-complex and CK MDS in large part overlap [9]. The TP53-complex MDS group comprises about 10% of total MDS; in this group, there are 74% multi-hit *TP53* and 26% mono-hit *TP53*; 91% of multi-hit *TP53* MDS had CK [9]. The CK group can be subdivided into two subgroups: one with *TP53* mutations and with del(5q) in 87% of cases, and the other without *TP53* mutations and with del(5q) in 32% of cases [9]. The presence or not of *TP53* mutations subdivides MDS with CK into two prognostic subgroups [9].

Huber and colleagues have analyzed a group of 789 MDS harboring del(5q); these MDS patients were extensively characterized at the molecular level, and 42% had isolated del(5q), 50% had CK, and 8% were not classified as CK or MDS del(5q) [10] (Figure 1). *TP53* mutations were detected in 54% of cases, with a higher frequency among patients with CK compared to those without CK (87% vs. 20%, respectively), and were also more commonly multi-hit among MDS-CK compared to MDS del(5q) (81% vs. 27%, respectively) [10]. Interestingly, the analysis of clonal hierarchy of del(5q) and *TP53* mutations showed that the VAF of del(5q) (considered as a measure of cytogenetic del(5q) clone size comparable to VAF of mutations) was similar in both MDS del(5q) and MDS-CK, while the VAF of *TP53* mutations was lower in MDS del(5q) than in MDS-CK [10]. These observations support a different hierarchical clonal origin of the two groups of MDS harboring del(5q): in MDS del(5q), the ancestral event is predominantly del (5q), while in MDS-CK, it is prevailingly a *TP53* mutation [10]. However, in a consistent number of cases, it is impossible to determine a hierarchy due to the similarity of the VAF of del(5q) and *TP53* mutations. In this study, a subset of 84 patients with MDS-del(5q) was analyzed in their evolution over time with a median follow-up of 2.8 months. A total of 4% of patients displayed a normal karyotype following transplantation or lenalidomide therapy; 4% of patients displayed clonal evolution with the acquisition of one additional chromosomal abnormality; 24% developed CK (with a median time to progression of 2.8 years for patients receiving lenalidomide treatment and 1.1 years without this treatment) and 69% showed a stable karyotype [10]. The acquisition of *TP53* mutations was more frequent among patients who developed CK compared with those who retained a stable karyotype (65% vs. 38%, respectively) [10].

## 3. Classification of MDS Associated with del(5q)

**Figure 2:**
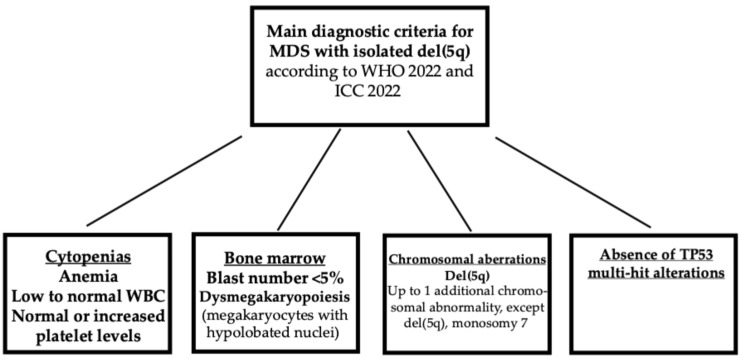
Main diagnostic criteria for MDS with isolated del(5q).

As discussed above, MSAs associated with 5q deletion must be subdivided into two different groups: one in which del(5q) is an isolated chromosomal abnormality and a second in which del(5q) is associated with other chromosomal abnormalities in the context of a complex karyotype (Figure 2).

According to the current ICC and WHO classifications of MDS with isolated del(5q), MDSs are defined not only according to the presence of an isolated del(5q) chromosomal abnormality and the absence of chromosome 7 abnormalities or CK, but also by a number of blasts <5% [3,4].

The taxonomy study showed that 22% of patients classified as del(5q) displayed a number of bone marrow blasts of >5% and should be excluded from this group according to ICC and WHO classifications [3,4] (Figure 2). However, the molecular taxonomy study proposes new criteria, where the driving and characterizing genetic alterations characterize a MDS group, and the blast frequency represents a measure to evaluate the extent of disease stages between different molecular groups and within the same molecular group [5]. Thus, according to this view, MDS-del(5q) with blasts <5% represents an earlier stage and MDS-del(5q) with blasts between 5 and 19% a more advanced stage of disease evolution [5]. In this group of patients, mOS was inversely correlated with blast cell counts in that MDS patients with del(5q) with 5–10% or >10% bone marrow blasts have a significantly shorter mOS compared to those with <5% of blasts [5]. These observations are in line with a study by Kewan et al., proposing a classification of MDS based on molecular patterns identified using a machine learning approach; this approach identified 14 molecularly distinct clusters and was not dependent on BM blast cell counts [11]. The observations made in many of some of these clusters showed a consistent intra-cluster heterogeneity of BM blast cell numbers, seemingly reflecting the stage of the disease rather than the molecular architecture [11].

Approximately 20% of MDS-del(5q) eventually progress to AML. Since the criteria of MDS-del(5q) of WHO 2022 and ICC 2022 exclude the presence of >5% of BM blasts and of adverse cytogenetics, current prognostic scoring systems, such as the Revised International Prognostic System (IPSS-R) and its molecular counterpart (IPSS-M), fail to efficiently stratify the risk of MDS with isolated del(5q) [12,13]. The new score system for MDS-del(5q) was built as a weighted sum of six prognostic variables, including hemoglobin, sex, number of mutations, *SF3B1* mutations and *TP53* multi-hit/*TP53* mono-hit with VAF >20% [14]. Using an IPSS-del(5q) score, a group of 682 MDS-del(5q) patients was stratified as low/very-low risk (51.6%, with an EFS of 78.2 months), intermediate (31.3% with an EFS of 45.1 months) and high-risk (17.1%, with an EFS of 28.2 months) [14].

The International Consortium for Myelodysplastic Syndromes proposed a harmonized classification system for MDS, representing an evolution of WHO 2022 and ICC 2022 [14]. The new classification identified nine clusters with different genomic features. One of these clusters was represented by MDS with isolated del(5q). The criteria for appurtenance to this cluster were represented by the presence of del(5q), absence of −7/del(7q) or CK, absence of biallelic *TP53* mutation and bone marrow blasts <5% [15]. It is important to note that 88% of MDS patients clusterized according to the above-mentioned criteria, excluding blast cell number, have bone marrow blasts <5%; the remaining 12% have blood number >5%, but were shifted in other clusters, such as in the cluster characterized by *TP53* mutations [15].

The identification, definition and prognostic stratification of the other group of MDS patients bearing del(5q) is largely related to the association with CK and *TP53* mutations. A fundamental study by Bernard et al. reported the molecular analysis of 3234 MDS patients, including 3787 *TP53*-mutated patients: 72.5% with a single mutation, 26.5% with two mutations and 1% with three mutations; allelic imbalance (due to focal deletions or regions of cnLOH) was observed in 178 patients [16]. According to the combination of mutations and allelic imbalances, four groups of patients were observed: Monoallelic mutation (33% of total *TP53*-mutated), multiple mutations without deletion of cnLOH affecting the *TP53* locus (24%); mutations and concomitant deletions (22%); and mutations and concomitant cnLOH (21%) [16]. In subgroups 2 to 4 of patients, there was a multi-hit *TP53* state (67%), while the mono-hit *TP53* state was observed in group 1 (33%); mono-hit cases were enriched in subclonal distribution (mVAF 13%), while multi-hit cases were predominantly clonal (mVAF 33%) [16]. Associations with complex karyotype, few co-occurring mutations, high-risk presentation and poor outcomes were specific to multi-hit patients; multi-hit status predicted a high risk of leukemic transformation [16]. Monoallelic *TP53* MDS patients did not differ significantly from *TP53*-WT patients [16].

Stengel et al. have analyzed the interplay of *TP53* allelic state, blast count, and complex karyotype on outcomes of MDS and AML patients [17]. This analysis showed that *TP53*-mutant MDS patients can be subdivided into three groups according to blast cell counts: <5% (24% with multi-hit *TP53* alterations), 5–10% (67% multi-hit *TP53* alterations) and 5–19% (91% multi-hit *TP53* alterations, with a mOS of 17, 10 and 8 months, respectively [17]. In patients with single-hit *TP53* alterations, the presence of CK considerably worsened mOS (46 months without CK vs. 14 months with CK) [17]. A more recent analysis showed that karyotypic clonal fraction (evaluated as low ≤50% clonal cells and high with ≥50% clonal cells) and the presence of CK are determinant factors for predicting adverse outcome of *TP53*-mutant MDS [18].

Shah et al. reported the analysis of 580 myeloid neoplasia patients (mostly MDS) and explored the impact of VAF percentage, *TP53* hit status, blast percentage and cytogenetic features on outcome [19]. Hierarchical analysis identified four risk groups with different survival rates: MDS-LB (low blast); MDS-EB1-EB2/AML VAF < 10%; MDS-EB1-EB2 > 10%; and AML VAF > 10% [19]. The significance of biallelic *TP53* status was limited to MDS < 5% of blasts and not extended to those with higher blast cell percentages; MDS-EB1 and -EB2 with VAF > 10% had comparable survival; MDS EB-1 and EB-2 with VAF < 10% and CK had a poor survival compared to those without CK and comparable to that observed for MDS EB-1 or EB-2 with VAF > 10% [19]. The frequency of MDS-LB and monoallelic *TP53* alterations with CK is markedly higher in cases with VAF > 10% compared to those with VAF < 10% [19].

## 4. Molecular Pathogenesis of MDS-del(5q)

The molecular pathogenesis of MDS-del(5q) is mainly related to the regions of the long arm of the deleted chromosome 5 (Figure 1). Boultwood and colleagues identified two commonly deleted regions (CDR): a distal 1.5 Mb deletion encompassing 5q32 and 5q33 (CDR1), associated with MDS-del(5q) and a better prognosis; and a proximal CDR at 5q31 (CDR2), associated with other types of MDS and AML, with CK and with worse prognosis [20,21,22]. It was estimated that the proximal CDR contains about 30 genes, while the distal CDR contains about 41 genes: CNV studies indicate 405 genes in the 5q region, 41 in the proximal CDR and 55 distal CDR. G-banding analyses, combined with FISH studies, showed that most of the deletions were large, extending from 5q13 to 5q33; the detailed characterization of 16 MDS-del(5q) patients showed a deletion of both CDRs in 15 of the 16 cases reported [21]. The study carried out by Jerez et al. showed a relevant role in MDS-del(5q) of some regions of 5q that are retained in the deletion process, called commonly retained regions (CRR): CRR1 for the proximal region, encompassing 81.7 Mb and ending at band 5q 14.2, and CRR2 for the distal region (5q34) [23]. CRRs are not observed in other MDS subtypes or AML with chromosome 5 deletions. Non-isolated del(5q) MDS showed NPM1/5q 35.1 monoallelic loss in 42.5% of cases versus 2.3% in isolated MDS-del(5q); gross chromosome abnormalities and monosomies, as observed in high-risk MDS, were significantly related to *NPM1* haploinsufficiency [24]. In non-isolated del(5q) MDS, centromeric breakpoints were significantly more frequently proximal to 5q14 than in cases of isolated del(5q) [25].

Chromosome analysis of the AML cases with isolated del(5q) showed a heterogeneous distribution of breakpoints involving del(5q); all AML cases with isolated del(5q) had deletions involving both CDRs at 5q31 and 5q33, and 7/12 cases of AML with isolated del(5q) had deletions extending into the distal CRR beyond 5q33 [24]. The most common breakpoint in AML with del(5q) is del(5)(q22q35) [26]. Interestingly, in AML as well, there are two groups of AMLs with del(5q) with distinct biologic features: one group with isolated del(5q), associated with frequent *IDH1* or *IDH2* mutations and with less frequent *TP53* mutations; and a second group with del(5q) observed in the context of CK and characterized by frequent *TP53* mutations [26].

The type of structural abnormalities of del(5q) is different in MDS with isolated del(5q) and in MDS with del(5q) associated with CK. These differences are related to the size of the 5q-deleted regions and to the eventual translocation of the deleted fragment of chromosome 5. Thus, in MDS patients, the size of the 5q deleted region was associated with the presence of *TP53* mutations and of additional chromosomal alterations [27].

The analysis of structural abnormalities of the 5q chromosome in MDS and AML patients showed two types of abnormalities: interstitial deletions and 5q loss due to unbalanced rearrangements. In unbalanced rearrangements, parts of deleted chromosome 5q were fragmented and inserted elsewhere in the genome, the most recurrent partners being chromosomes 17, 3, 7 and 18 [28]. The unbalanced translocation der(5,17) involving chromosome 5q and 17 is a recurrent aberration in MDS and AML, resulting in *TP53* loss; no fusion genes resulted from the unbalanced translocation [29]. This translocation is frequently observed in MDS-CK and AML-CK [29].

The analysis of a large cohort of MDS and AML patients with 5q deletions allowed us to evaluate the frequency of the loss of the long arm of chromosome 5 due to unbalanced rearrangements [30]. Unbalanced rearrangements occurred more frequently in AML than in MDS (45.6% vs. 32.8%, respectively) and were more frequent in MDS-CK than in MDS with isolated del(5q) (95.2% vs. 32.2%, respectively) [31]. Furthermore, chromosome 5 unbalanced rearrangements were associated with *TP53* mutations and shorter OS [30].

The pathogenesis of MDS-del(5q) seems to be related to a condition of haploinsufficiency involving the genes located in the chromosome 5q deleted regions. In line with this hypothesis, gene expression analysis of CD34^+^ cells derived from MDS-del(5q) patients showed that most of the genes located at 5q32-q33 (CDR1) displayed reduced expression levels [31].

Haploinsufficiency (HI) resulting from deletion of regions of the long arm of chromosome 5 and the accompanied loss of heterozygosity (LOH) are key pathogenic events in MDS-del(5q). Adema et al. analyzed genomic profiles at the level of gene alterations and gene expression and clinical phenotypes of 388 myeloid neoplasms (mostly MDS) with del(5q) [32]. The analysis of clonal architectures of MDS with deletions of 5q showed that not in all cases del(5q) is a primary hit; in fact, in some MDS, del(5q) is preceded by other mutations, such as *TP53* mutations; in other MDS, del(5q) is codominant with *TP53* mutations [32]. When del(5q) is dominant, in isolated MDS-del(5q), mutations in *CSNK1A1* are the most common secondary hit, while in CK-MDS with del(5q), *TP53* alterations are the primary hit [32]. The analysis of the genes involved in the deletion events at the level of 5q allowed for differentiating these genes at the functional level into HI-driver genes, which provide support to promote clonal growth, and HI-anti-driver genes promoting the phenotypic dysplasia and apoptosis of del(5q) [32]. HI-driver genes are *CSNK1A1*, *CTNNA1* and *TCERG1*. HI-anti-driver genes include *RPS14*, *HSPA4*, *SIL-1* and *UBE*_2_*D*_2_, all promoting increased apoptosis of MDS-del(5q) cells. The balance between the effects of HI-driver and HI-anti-driver genes determines the growth features of MDS-del(5q). The growth of MDS-del(5q) is dictated by the capacity of some HI-drivers, which provide the triggering for MDS clonal expansion, to enable a selective process to induce mechanisms suitable to overcome the pro-apoptotic effect of HI-anti-driver genes. This selective pressure triggers the acquisition of accelerator events, such as *CSNK1A1* mutations, *TP53* alterations and/or monosomy 7 [32].

The CDR is rich in genes functionally relevant, homing 40 genes, 33 of which were expressed in HSC/HPC CD34^+^ cells [22].

The haploinsufficiency of the various genes located in the 5q deleted regions is responsible for the phenotypic features of MDS-del (5q). (Table 1)

### 4.1. RPS14

Anemia refractory to standard treatments is one of the typical features of MDS-del (5q). The development of anemia in these patients seems to be related to haploinsufficiency and consequent reduced expression of *RPS14*. The *RPS14* gene maps to chromosome 5q33.1 and encodes a ribosomal protein that is a component of the 40S ribosome subunit. The expression of *RPS14* was significantly reduced in CD34^+^ cells of MDS-del (5q) patients [31]. Ebert and colleagues showed through RNA interference experiments that deficient expression of RPS14 in MDS-del(5q) was responsible for the development of anemia; enforced expression of RPS14 rescues the disease phenotype in patient-derived bone marrow cells [33]. In bone marrow cells of MDS-del(5q) patients and in animal models, the impaired formation of the 40S ribosomal subunit induces an upregulation of the p53 pathway in erythroid cells [34,35,36].

Haploinsufficiency of *RPS14* in patients with MDS-del(5q) syndrome is associated with deregulated expression of ribosomal and translation-related genes, suggesting that 5q^−^ syndrome represents a disorder of aberrant ribosome biogenesis [37].

Pellagatti et al. showed that haploinsufficiency of *RPS14* and deregulation of ribosomal- and translation-related genes were equally observed in MDS with del(5q) associated with CK [38].

RPS14 deficiency in MDS is not limited only to MDS-del(5q) but is also observed in some MDSs not associated with del(5q). In fact, a subset of low-risk MDS patients, without 5q deletion and *RPS14* gene mutations, displays a significantly low expression of *RPS14* [38]. In these patients, low *RPS14* expression was associated with a better prognosis compared to low-risk MDS patients with normal *RPS14* expression [39]. The survival of non-5q MDS patients with low *RPS14* expression seems to be improved by immune-modulating drugs, thus suggesting that these MDS symptoms may improve in MDS-del(5q) patients being administered lenalidomide [40,41]. Adema et al. reported the genomic and expression profiles of 995 MDS patients, 170 with MDS-del(5q) and 825 with diploids for 5q; as expected, MDS-del(5q) patients displayed haploinsufficiency, associated with reduced expression of *RPS14* [42]. The non-del(5q) NDS were grouped in six different clusters; cluster 2 displayed a normal karyotype, frequent *ASXL1* and *TET2* mutations, and marked downregulation of *RPS14* expression in all the patients included in this cluster [42]. However, an important difference between these two groups of MDS with low *RPS14* expression is that only MDS-del(5q) displays frequent *TP53* alterations and *CSNK1A1* mutations [42].

Schneider et al. generated mice with conditional inactivation of *RPS14* and showed, in these mice, a defect in erythroid differentiation dependent upon p53 activation and consisting of apoptosis occurring at the transition from polychromatic to orthochromatic erythroblasts [43]. This defect was responsible for the development of a progressive anemic condition, megakaryocyte dysplasia, and low levels of hematopoietic stem cell quiescence [43].

### 4.2. miR-145 and miR-146a

A typical feature of MDS-del(5q) is represented by normal or increased platelet levels, megakaryocyte hyperplasia associated with small, hypolobated and dysmorphic bone marrow megakaryocytes (many having a plasmocytoid appearance). These features seem to be related to the deletion of two miRNAs, miR-145 and miR-146a, located in the 5q-deleted region in 5q^−^ syndrome [44]. The study of animal models showed that the loss of miR-145 and miR-146a induces dysmegakaryopoiesis, thrombocytosis and innate immune signaling [44]. Other studies showed that in mouse models, the combined loss of miR-146 and RPS14 induces the generation of abnormalities of megakaryocytic differentiation similar to those observed in MDS-del(5q) patients [45].

### 4.3. CSNK1A1

MDSs originated from the initial growth and clonal expansion of a hematopoietic stem cell (HSC), which acquired a somatic gene alteration conferring a selective growth advantage over normal HSCs. The study of some genes involved in del(5q) suggests that their haploinsufficiency could contribute to the proliferation and expansion of del(5q)-mutant hematopoietic cells. The reduced expression of the tumor suppressor gene Casein Kinase 1A1 (CSNK1A1) seems to play a relevant role in conferring a growth advantage to del(5q) cells, promoting their clonal expansion. The study of a murine model with conditional inactivation of *CSNK1A1* showed that haploinsufficiency induced HSC expansion and a competitive repopulation advantage, whereas homozygous deletion induced HSC failure [46]. *CSNK1A1* mutations occurring in the non-deleted allele occur frequently in MDS-del(5q) patients. Smith and colleagues retrospectively analyzed 250 MDS-del(5q) patients and observed that 16% of these patients had *CSNK1A1* mutations, all missense mutations and occurring in a region of this protein highly conserved and involved in ATP catalysis; the presence of *CSNK1A1* mutations was associated with reduced response to lenalidomide [47]. In total, 57% of patients carrying a *CSNK1A1* mutation showed disease progression coupled with an increased allele burden [47]. Heuser et al. reported *CSNK1A1* mutations in 7.2% of MDS-del(5q) patients, all missense mutations occurring either at the level of glutamic acid E98 or at the level of aspartic acid D140; the presence of *CSNK1A1* mutations was associated with significantly reduced OS compared to MDS-del(5q), *CSNK1A1*-WT patients [48]. Stolman and colleagues used a genetic barcoding strategy to compare genes implicated in the pathogenesis of MDS-del(5q) in direct competition with each other and with WT cells and showed that *CSNK1A1* haploinsufficient HSCs expand clonally and compete with all other tested genes and combinations [49]. In mouse models, a cooperation between *CSNK1A1* haploinsufficiency and *TP53* mutations was observed in promoting clonal advantage and leukemic transformation through MAPK and MYC pathway activation [50].

*CSNK1A1* haploinsufficiency in MDS-del(5q) leads to increased platelet counts, while recurrent somatic mutations of *CSNK1A1* within the del(5q) CDR in MDS determine a homozygous *CSNK1A1* defect with concurrent thrombocytopenia in the affected patients [46]. In line with these observations, a recent study showed that CRISP3/Cas9-mediated genetic ablation of *CSNK1A1* in human megakaryocytes resulted in a substantial defect in megakaryocyte maturation and platelet production [51]. These observations support an important biologic role of *CSNK1A1* in megakaryocyte differentiation and maturation, being required for cytoskeletal dynamics and polarization, in addition to proplatelet formation and polyploidization [51].

Mutations in *CSNK1A1* have been observed at the level of E98 (E98K) and D140 (D140A); these mutants are the two most frequently observed in MDS-del(5q). E98K and D140A mutants have a reduced capacity to promote phosphorylation of beta-catenin, thus inducing enhanced Wnt signaling; furthermore, E98K and D140A mutants displayed enhanced binding to the p53 inhibitor MDMX, increased MDMX-p53 binding and increased suppression of p21 expression [52]. These functional changes induced by CSNK1A1 mutants promote expansion of abnormal myeloid progenitors in MDS-del(5q).

### 4.4. HSPAP9 and SPARC

The haploinsufficiency of heat shock protein A9 (HSPA9) also seems to contribute to the erythroid maturation defect observed in MDS-del(5q). The HSPA9 gene encodes a protein called mortalin and is located in the 5q31.2 region (proximal CDR); this protein belongs to the HSP70 family and plays a role in various biological processes, including control of cell proliferation, response to cell stress, and inhibition of apoptosis [53]. Knockdown of HSPA9 in human hematopoietic cells significantly delayed the maturation of erythroid precursors, but not of myeloid or megakaryocytic precursors [54]. Knockdown of HSPA9 in a murine bone marrow transplantation model resulted in a decrease in hematopoietic progenitors, including a decrease in erythroid precursors [54]. Other studies have shown that knockdown of HSPA9 in human CD34^+^ cells induces apoptosis of HPCs Via TP53 activation [55]. More recently, there has been evidence that inhibitors of HPSA9 expression in human CD34^+^ cells resulted in an increased expression of TP53 in these cells and in a block of erythroid maturation; this block in erythroid maturation was in part inhibited by knockdown of TP53 [55]. These observations suggest that the reduced levels of HSPA9 expression may contribute to the anemia commonly observed in MDS-del(5q) patients [56].

The gene encoding secreted protein, acidic and rich in cysteine (SPARC), is located on human chromosome 5 at the level of 5q32; due to haploinsufficiency, its level of expression is significantly decreased in CD34^+^ cells of MDS-del(5q) patients [57]. The study of SPARC-null mice showed a hematologic phenotype, characterized by thrombocytopenia and reduced numbers of early erythroid progenitors (BFU-E) [57]. Another study confirmed that SPARC expression is required for the development of erythroid progenitors, but not for erythroid maturation [58].

### 4.5. CTNNA1

Liu et al. have explored the expression in MDS-del(5q) of 12 genes present at the level of CDRs and normally expressed in HSCs, as well as the analysis of the expression of these genes in leukemia-initiating stem cells of MDS-del(5q) patients [59]. Among these genes, the gene encoding α-catenin (*CTNNA1*), located at 5q32.1, is expressed at markedly lower levels in leukemia-initiating cells from MDS or AML patients with del(5q) than in AML or MDS patients without del(5q) or in normal HSCs [59]. Analysis of the gene promoter of the *CTNN1A* normal allele in del(5q) leukemic cells showed gene expression inhibition by methylation and histone deacetylation [59]. The loss of expression of α-catenin provides a growth advantage to AML or MDS cells with del(5q) [59].

### 4.6. EGR1

The *EGR1* gene encodes the transcription factor EGR1, located at the level of 5q31, a region frequently deleted in MDS and AML with del(5q). Studies of murine models with heterozygous or homozygous deletions of the *EGR1* gene support an important role of this gene in the control of hematopoiesis and as a tumor suppressor gene. *EGR1^−/−^* mice showed elevated white blood cell counts, elevated lymphocytes, decreased neutrophil counts and an incapacity to maintain normal RBC counts [60]. *EGR1^−/−^* or *EGR1^−/+^* mice treated with phenylhydrazine develop anemia and are unable to be cured of their anemic condition [60,61]. *EGR1^−/−^* mice treated with the DNA alkylating agent, N-ethyl-nitrosourea, develop immature T-cell lymphomas or myeloproliferative disorders, characterized by elevated WBC, anemia with ineffective erythropoiesis and thrombocytopenia [61].

EGR1 binds genes critical for stem cell differentiation, inflammatory signaling, and the DNA damage response [62]. Haploinsufficiency of *EGR1* biases HSCs/HPCs toward a self-renewal transcriptional signature, characterized by upregulation of MYC-driven proliferative signals, downregulation of p21, disrupted DNA damage response, and downregulated inflammation [62].

### 4.7. CDC25 and PP2A

Dual-specificity phosphatases cell division cycle 25 (CDC25) and protein phosphatase-2 (PP2) are encoded by genes located at 5q 31.2, a CDR in MDS-del(5q). Both these phosphatases are regulators of the cell cycle G_2_-M transition. Gene expression studies showed that CDC25 and PP2 expression is significantly reduced in MDS-del(5q), compared to MDS-5q-WT and normal controls [63]. Haploinsufficiency for *CDC25* and *PP2A* genes does not seem to be involved in the generation of the peculiar hematologic phenotype of MDS-del(5q) but is essential for promoting selective sensitivity of MDS-del(5q) to lenalidomide-induced apoptosis [63]. Treatment of del(5q) leukemic cells with lenalidomide induces G_2_ arrest and apoptosis, whereas there was no effect in non-del(5q) leukemic cells [62]. Small interfering RNA suppression of *CDC25* and *PP2A* gene expression recapitulates del(5q) susceptibility of MDS-del(5q) cells to lenalidomide with G_2_ arrest and induction of apoptosis [64].

### 4.8. DELE1

The Death Ligand Signal Enhancer (*DELE1*) gene is located in the 5q31.3 region and encodes a protein associated with the inner mitochondrial membrane and is involved in death receptor-mediated apoptosis. *DELE1* is one of these whose expression is most under-expressed in MDS-del(5q) [65]. Recent studies have shown that DELE1 protein is involved in the relay of mitochondrial stress to the cytosol through the OMA1-DELE1-HRI pathway, which leads to the activation of ATF4, the master transcription factor of the integrated stress response [63]. Partial loss of *DELE1*, as observed in MDS-del(5q) patients, was sufficient to reduce the sensitivity to mitochondrial stress in leukemic cells [65].

### 4.9. DIAPH1

Formins are highly conserved proteins involved in the assembly of actin microfilaments and microtubule cytoskeletons into cell architectures able to support cell adhesion and migration. The mammalian diaphanous-related formins are encoded by DIAPH genes; the three *DIAPH* gene isoforms are encoded by the *DIAPH1* gene, located on chromosome 5q31.3. *DIAPH1*-deficient mice (in mice defined as *mDia1*) develop age-dependent myeloproliferative or myelodysplastic phenotypes, suggesting that *DIAPH1* may act as a tumor suppressor [66]. The study of mice *mDia1* heterozygous or homozygous showed that *mDia1* deficiency led to a cell-autonomous overexpression of the membrane antigen CD14 and a hypersensitive innate immune response mediated by CD14/TLR4-like signaling; these mice develop age-dependent MDS that is accelerated by chronic stimulation of innate immunity [67].

An important role of DIAPH1 in megakaryocyte proplatelet formation through the accumulation of the actin and microtubule cytoskeletons was shown in another study [68].

A recent study reported that the frequent occurrence of *DIAPH1* mutations in these patients was correlated with lower megakaryocyte dysplasia in low-risk patients and higher megakaryocyte counts pre-transplant [67]. *GP1BA* and *SETB1* mutations were positively and negatively associated with *DIAPH1* mutations, respectively [69]. *DIAPH1*-mutated patients showed a favorable outcome [69].

### 4.10. TIFAB

TRAF-interacting proteins with forkhead-associated domain B (TIFAB) are a TIFA family homolog lacking a phosphorylation site and a TRAF6 motif, acting as a negative regulator of TIFA-TRAF6 signaling. Given these biochemical effects, TIFAB acts as an inhibitor of NF-kB signaling. The *TIFAB* gene is located within the proximal CDR on band 5q31.1; consistent with haploinsufficiency, expression of TIFAB is decreased by about 50% in MDS-del(5q) compared to MDS without del(5q) or normal bone marrow [70]. Gene knockout studies provided evidence that TIFAB displays tumor suppressor-like functions, and its deletion induces an MDS-like phenotype in mice by modifying the dynamic range of the immune pathway reactivity in HSCs [71]. Verney et al. explored the mechanisms through which loss of *TIFAB* affects hematopoiesis: *TIFAB* loss increases TRAF6 protein levels and the dynamic range of TLR4 (Toll-like receptor 4) signaling, contributing to ineffective hematopoiesis [72]. This effect of TIFAB is potentiated by the concomitant loss of *miR-146a* [72].

Another study showed that TIFAB regulates ubiquitin-specific peptidase 15 (USP15) and consequently the USP15-mediated p53 signaling [73]. Deregulation of the TIFAB-USP15 complex, as observed in MDS-del(5q), modulates p53 activity and has critical functional consequences for HSCs under stress conditions [73].

### 4.11. NPM1

Nucleophosmin 1 is the most frequently mutated gene in AML; this gene is located on chromosome 5q35 and is lost in about 10% of MDS arising from large 5q deletions, mainly occurring in MDS with CK and in t-MDS [23,24]. *NPM1^+/−^* mice display an increased susceptibility to leukemia development and have been shown to generate hematologic syndromes with features similar to human MDS [74]. A mouse model of *NPM1* knockout in HSCs showed premature aging of HSCs and increased inflammatory response, which favors the development of an MDS-like condition [75]. *TP53* loss exacerbates the leukemic transition of *NPM1*-KO HSCs [75].

## 5. MDS-del(5q) as a Contiguous Gene Syndrome

The observations above suggest that MDS-del(5q) is a contiguous gene syndrome in which haploinsufficiency for several genes contributes to the hematologic phenotype observed in these patients. Some studies have explored whether the combined haploinsufficiency of several genes located on 5q and deleted in MDS-del(5q) induces a hematologic phenotype comparable to that observed in these patients. Thus, Ribezzo et al. have explored the combinatorial effect of haploinsufficiency for *RPS14, SSNK1A1* and *miR-145* using mice with genetically engineered, conditional heterozygous inactivation of *RPS14* and *CSNK1A1* and stable knockdown of *miR145/mir146a* [76]. These mice recapitulated all the main phenotypic features of MDS-del(5q) patients, including severe anemia, dysmegakaryopoiesis with typical morphological abnormalities and an increase in innate immune functions in macrophages (reflected by decreased phagocytic function and increased expression of S100A8) [76].

Another experimental model of MDS-del(5q) was based on a mouse model in which *TIFAB* and *miR-146a* were simultaneously deleted [77]. This model recapitulates several aspects of MDS-del(5q), such as defects of HSCs/HPCs mainly affecting the myeloid lineage, progressive peripheral blood cytopenias, myeloid dysplasia and altered cytokine production in the bone marrow microenvironment [77]. Deletion of *TIFAB* and *miR-146a*, as observed also in MDS-del(5q), induces the activation of IRAK2 and TRAF6, which determines an aberrant function of HSCs/HPCs. The study of *TIFAB^−^/mir-146a^−^* mice showed an impaired capacity to respond to an inflammatory condition, with a reduced number of HSCs/HPCs and increased p53 expression [78]. This reduced proliferative capacity of HSCs/HPCs was restored by *TP53* inactivation, thus indicating that inflammation confers a competitive advantage to functionally defective del(5q) cells upon loss of *TP53* [78]. Thus, increased p53 activation in MDS-del(5q) HSC/HPCs due to inflammation triggers a selective pressure for genetic inactivation of p53 or expansion of pre-existing *TP53*-mutant clone [78].

## 6. Therapy-Related MDS-del(5q)

Therapy-related MDS (t-MDS) is defined as MDS occurring after exposure to chemotherapy and/or radiation therapy and corresponds to about 15–20% of all MDS. T-MDS, compared to primary MDS (p-MDS), is characterized by higher-risk clinical features, including more cytogenetic aberrations, higher frequency of high-risk mutations and a shorter overall survival. A nation-wide study confirmed that t-MDS displays a significantly shorter mOS compared to p-MDS (15.8 months vs. 31.1 months, respectively) [79].

Studies on large cohorts of t-MDS patients compared to p-MDS provided evidence about significant differences in their mutational profiles, such as *TP53* and *PPM1D* mutations being clearly more frequent in t-MDS than in p-MDS; and *ASXL1*, *TET2*, *SRSF2* and *SF3B1* mutations being less frequent in t-MDS than in p-MDS [12,80,81]. T-MDS had a shorter mOS than p-MDS even in patients who received allo-HSCT [81].

Chromosome 5 abnormalities are frequent in t-MDS, being observed in about 40% of patients [82]. However, isolated del(5q) was observed at a similar frequency in t-MDS and p-MDS [79].

The frequency of del(5q)/monosomy 5 abnormalities and of CK was significantly higher in t-MDS compared to p-MDS (for del(5q)/−5, 30% vs. 14% and for CK, 28% vs. 11%, respectively) [83]. Median survival for t-MDS patients was significantly shorter than for p-MDS within all risk group categories [83]. Furthermore, patients with t-MDS had a significantly higher hazard of death relative to p-MDS [83].

Hiwase et al. explored a group of 377 patients with therapy-related myeloid neoplasms (65% of t-MDS and 35% of t-AML) and observed that 34% of these tumors harbor *TP53* mutations [84]. The frequency of chromosome 5 abnormalities (del(5q)/monosomy 5) was markedly higher in *TP53*-mutant MDS (81.4 in multi-hit and 66.7% in mono-hit) than in *TP53*-WT tumors (4.3% in *TP53*-WT); the same applied to CK (88.6% vs. 11.5%) [84]. The analysis of the mOS in t-MDS patients showed that *TP53*-mutant patients had a significantly shorter survival than *TP53*-WT patients; among *TP53*-mutant MDS, neither the allelic status nor the bone marrow percentage provides a significant prognostic information; and 10% *TP53* VAF is a clinically useful threshold to identify patients with poor survival [84].

In another study, Shah et al. have reported the analysis of a group of 488 t-MN patients (65% t-MDS and 35% t-AML), showing that 37% of these patients display *TP53* alterations (multi-hit in 88% of cases) [85]. In these patients, del(5q) was observed in most cases in association with *TP53* alterations [85]. The proportion of *TP53* mutations increases from 4.5% in patients with a normal karyotype to 17.3% in cases with two chromosomal abnormalities and 76.8% in cases with CK [85]. Importantly, the enrichment of *TP53*-mutant was observed in cases with del(5q) without CK; such enrichment of *TP53* mutations was not observed in cases with del(7q) without CK [85]. These findings support the view that *TP53* mutation burden increases not only with the number but also the type of chromosomal abnormalities [85]. *TP53*-mutant VAF ≥ 10% MDSs are associated with distinct presentation, profile of genomic instability and outcomes [85].

Buo explored a group of 138 t-MDS. 33% of these patients displayed *TP53* abnormalities (73% multi-hit and 27% mono-hit) [86]. Del(5q) was observed in 30% of t-MDS compared to 10.9% in a group of p-MDS: CK in 39.2% of t-MDS and 15.7% of p-MDS [86]. In the group of t-MDS, del(5q)/−5 was observed in 57.5% of *TP53*-mutant and 17.8% of *TP53*-WT, while CK was observed in 85% of *TP53*-mutant and 18.9% of *TP53*-WT [86]. In t-MDS, del(5q)/−5 was similarly associated with multi-hit or single-hit *TP53* alterations (60% vs. 50%, respectively) [86]. In t-MDS *TP53*-mutated, 100% of MDS with del(5q)/−5 are associated with CK, while in *TP53*-WT 72% of MDS with del(5q)/−5 are associated with CK [86]. In t-MDS, *TP53*-mutant MDS all none of the MDS with del(5q) display an isolated del(5q), while 28% of *TP53*-WT MDS display isolated del(5q) [86]. It is of interest to note that t-MDS exhibit a markedly higher frequency of *TP53* and *PPM1D* mutations and a markedly lower frequency of *ASXL1*, *U2AF1* and *SRFSF2* mutations compared to p-MDS [86]. In t-MDS patients, it was significantly poorer for *TP53*-mutant compared to *TP53*-WT patients; among t-MDS, particularly *TP53*-mutant patients, mOS does not seem to be affected by *TP53* VAF or *TP53* multi-hit or mono-hit status [86]. *TP53* mutations in t-MDS are strongly associated with genomic instability; in fact, in *TP53*-mutant MDS, the frequency of patients with >3 cytogenetic abnormalities is very high, while in *TP53*-WT patients, it is low [86].

Lossard and colleagues explored the profile of chromosome abnormalities in 110 MDS-del(5q), 82 p-MDS and 28 t-MDS patients [87]. The breakpoints for 5q varied considerably in that the deletion size may be small (mainly 5q31), intermediate (with a size equivalent to half of the 5q arm) or large (corresponding to almost all the 5q arm) [87]. Among t-MDS patients, the frequency of small (21%), intermediate (18%) and large deleted fragments (61%) was similar to that observed for p-MDS patients [87].

As reported above, a minority of t-MDS displays a condition of isolated del(5q). Patients with isolated MDS-del(5q) show outcomes similar to those observed for p-MDS patients with isolated del(5q), for that concerns the response to lenalidomide treatment, mOS and the rate of leukemic transformation [88].

As discussed above, t-MDS can be subdivided into two groups with different mutational profiles and prognoses: one group characterized by consistent genomic instability with frequent *TP53* mutations and numerous cytogenetic abnormalities is associated with a poor prognosis; a second group with lower genomic instability with absent *TP53* mutations and a lower number of cytogenetic abnormalities is associated with a prognosis comparable to that of p-MDS. The longitudinal study of patients who developed t-MDS and t-AML suggests a pathogenetic mechanism based on the presence in these individuals of very minoritarian *TP53* mutant clones of HSCs/HPCs that preferentially expanded after exposure to chemotherapy [80]. This interpretation is supported by the study of murine bone marrow chimeras containing both WT and *TP53*^+/−^ HSCs/HPCs, the *TP53*^+/−^ HSCs/HPCs preferentially expanded after exposure to chemotherapy [80]. These observations suggest that cytotoxic therapy does not directly induce *TP53* alterations and that HSCs/HPCs bearing *TP53* mutations are resistant to chemotherapy and expanded preferentially after treatment [80]. The early acquisition of *TP53* mutations in the ancestral HSC/HPC clones could contribute to the accumulation of the frequent cytogenetic abnormalities.

## 7. Progression and Disease Evolution in MDSA-del(5q)

A high proportion of patients with isolated MDS-del(5q) respond to treatment with lenalidomide; however, 40% of these patients progress to AML by 5 years after starting treatment.

Several studies have documented treatment-emergent *TP53* mutations in patients with MDS-(del5q) receiving lenalidomide therapy. In a first case-report study, Jadersten et al. reported the case of a patient with MDS-del(5q) without *TP53* mutations at diagnosis, with complete erythroid and partial cytogenetic response to lenalidomide, who evolved to high-risk MDS with a complex karyotype, associated with *TP53* mutations [89].

Scharenberg and colleagues have reported the analysis of the progression of 35 MDS patients with isolated del(5q) treated with lenalidomide (22 patients) or not (13 patients) and analyzed them over time for various clinical and laboratory parameters, including targeted sequencing for the most relevant MDS-related mutations [90]. Progression was observed in 13 patients (4 with high-risk MDS and 9 patients with AML) and was associated with the detection of some new recurrent mutations, either occurring alone or in combination: *TP53* in 9 cases, *TET2* in 6 cases, *RUNX1* in 3 cases, *PTPN11* and *SF3B1* in 1 case [90]. *TP53* mutations were observed in seven out of nine patients progressing to AML [90].

Mossner et al. have explored the adaptation and evolution of mutational hierarchies of MDS patients undergoing treatment. In their study, they included 28 MDS patients with del(5q), of which 21 were defined as isolated MDS-del(5q) according to WHO criteria [91]. Molecular analysis showed del(5q) was acquired as a secondary lesion or constituted a minor independent clone in 54% of all del(5q) MDS and in 62% of those with isolated del(5q); del(5q) was a founder event alone in 21.4% of the cases and with other founder lesions in 28.5% of cases [91]. Thus, del(5q) appeared to be the founder lesion in some patients and the secondary hit in other MDS patients. The longitudinal follow-up of these patients during their treatment with lenalidomide showed different patterns of molecular evolution. Some patients displayed a dynamic evolution of branching, with disappearance upon lenalidomide treatment of initially dominant subclones carrying del(5q); despite a significant improvement of hematological parameters, these patients displayed rapid emergence of previously undetectable branching subclones with new aberrations subsequent to lenalidomide administration [91]. Other patients showed rapid oligoclonal turnover following lenalidomide treatment, with cytogenetic remission of their del(5q) bearing subclones, early founding clones expanded in bone marrow [91]. The same authors reported the frequent detection of *TP53* mutations in MDS-del(5q) with isolated 5q abnormality, negative for *TP53* mutations at diagnosis and treated with lenalidomide (6 out of 15 patients) [92]. The study of eight patients with *TP53* mutations before the start of treatment with lenalidomide showed the negative impact of *TP53* mutations on survival and lower sensitivity of *TP53*-mutant clones to lenalidomide treatment [92].

Lode and colleagues have reported the study of 24 MDS-del(5q) patients undergoing treatment, with 75% of patients reporting transfusion independence and 21% of complete cytogenetic responses [93]. In total, 25% of these patients displayed *TP53* mutations at diagnosis and 38% developed *TP53* mutations during follow-up [93]. A correlation was observed between the acquisition of *TP53* mutations and disease progression [93].

Sperling et al. reported the study of 416 patients who had developed therapy-related neoplasms (t-MN) and who had a detailed prior exposure history [94]. In these patients, *TP53* mutations were significantly associated with thalidomide analogs, particularly with lenalidomide [94]. In vitro and in vivo experimental studies supported that the effect of lenalidomide was specific to HSC/HPC with *TP53* mutations [94]. This selective advantage of *TP53*-mutant HSCs/HPCs was conferred by lenalidomide and not by other thalidomide analogs, such as pomalidomide, this difference being related to the capacity of lenalidomide but not of pomalidomide to induce the degradation of CSNK1A1 [94]. These observations have suggested that lenalidomide induces the expansion of pre-existing *TP53*-mutant clones that are less sensitive to the suppressive effects exerted by this drug.

Abdallah and colleagues reported the analysis of 10 Mayo Clinic patients with MDS-del(5q), analyzed by NGS before and after treatment with lenalidomide [95]. Two of these patients had *TP53* mutations at diagnosis, and three acquired *TP53* mutations (two of these patients also displayed *SF3B1* mutations at diagnosis) [95]. In the three patients acquiring *TP53* mutations after therapy, *TP53* mutations were monoallelic in one patient and biallelic in the other two patients [95]. The patients developing *TP53* mutations after therapy apparently did not display any peculiar characteristics compared to the rest of the patients [95].

Feurstein et al. have explored the routes of clonal evolution into CK in 1684 MDS patients with del(5q); 161 of these patients showed additional cytogenetic abnormalities that developed over time: 134 patients (8%) developed cytogenetic aberrations within the del(5q) clone and were defines as clonal evolution, while 27 patients developed independent clones not present within the del(5q) clone [96]. Two main pathways of cytogenetic clonal evolution have been identified: a more frequent (61% of cases) stepwise accumulation of cytogenetic events over time; a less frequent (39% of cases) catastrophic event, characterized by the occurrence of two or more aberrations occurring at the same time, determining the sudden development of clones bearing a CK [96]. The most frequent aberrations in the group with stepwise accumulation were trisomy 8 and trisomy 21; in the group with catastrophic events, del(7q)/−7 and del(17p)/−17 were the most recurrent chromosomal abnormalities [96]. Loss of 17p or monosomy 17 determines the loss of *TP53*, which could represent the driving force in MDS patients with del(5q) who undergo a sudden catastrophic event [96].

## 8. Treatment of MDS-del(5q)

The treatment of MDS-del(5q) is diversified according to the type of MDS associated with del(5q), being different for MDS with isolated del(5q) and for MDS with del(5q) associated with CK (Table 2).

Several recent studies have reviewed the treatment of low-risk MDS and, particularly, of MDS with isolated del(5q) [97,98,99]. Treatment goals for these patients include transfusion independence, an increase in hemoglobin level, improvement of survival and maintenance or improvement of quality of life. Anemia observed in MDS-del(5q) patients was shown to be highly responsive to treatment with lenalidomide. According to the results observed in several studies, including randomized clinical trials, the FDA and EMA approved lenalidomide for the treatment of low- or intermediate-1 risk MDS with del(5q) and up to one additional cytogenetic abnormality, excluding chromosome 7 abnormalities. Usually, MDS patients with isolated del(5q) have a good prognosis. However, the presence of co-occurring *SF3B1* or *TP53* mutations may worsen this prognosis. In fact, a study by Meggendorfer et al. showed that MDS patients with isolated del(5q) and co-occurring *SF3B1* mutations displayed a shorter OS compared to those without *SF3B1* mutations (50 months vs. not reached) [6]. On the other hand, Huber et al. characterized a group of 231 MDS patients with *SF3B1* mutations and observed that del(5q) and *RUNX1* mutations were independent prognostic factors for overall survival [100]. Furthermore, among *SF3B1*-mutant MDS, those associated with del(5q) exhibited a higher frequency of *TP53* and *RUNX1* co-mutations compared to the *SF3B1*-mutant MDS without del(5q) [100].

Other studies have confirmed that *SF3B1* co-mutations confer poor outcomes to MDS-del (5q). Thus, Chan et al. reported that MDS-del(5q) patients with *SF3B1* mutations had shorter mOS compared to WT (23.9 months vs. 83.5 months) [101].

Other studies have provided evidence that MDS with concurrent del(5q) and *SF3B1* mutations exhibit morphological, immunophenotypic and clinical properties mixed between those typically observed in MDS with isolated del(5q) and MDS with *SF3B1* mutations, such as ringed sideroblasts and thrombocytosis [102,103]. Furthermore, the risk of transforming to AML is also higher in patients with both del(5q) and *SF3B1* mutations compared to those with *SF3B1* mutations alone.

A recent study reported a retrospective analysis on 77 MDS-del(5q) patients with *SF3B1* mutations (*SF3B1^del5q^*); these patients received first-line treatment with ESAs (mostly with lenalidomide) [104]. The mOS of these patients was 66 months: 109 months for *SF3B1^del5q^*/*TP53*-WT and 64 months for *SF3B1^del5q^*/*TP53*-mutant [104]. mOS was compared with a group of MDS-*SF3B1* and MDS-del(5q) patients; mOS was 66,82 and 103 months, respectively, for *SF3B1^del5q^*, MDS-del(5q) and MDS-*SF3B1*, with a rate of AML transformation in these three groups of 20%, 12% and 5%, respectively [104]. These observations support the conclusion that the mOS observed for *SF3B1^del5q^* patients was inferior to either isolated del(5q) patients or MDS-*SF3B1* patients and may be driven by a higher rate of concurrent *TP53* and *RUNX1* mutations [104].

Although there was some controversy about the prognostic impact of *TP53* mutations in MDS with isolated del(5q), a large retrospective analysis carried out in 682 patients with isolated del(5q), defined according to Bernard et al. [6]; Montoro et al. showed that 18.9% of patients displayed *TP53* mutations: the majority of these patients were monoallelic for *TP53* alterations and only 4.5% with multi-hit *TP53* alterations [105]. Patients with the *TP53* monoallelic mutation with VAF >20% had a 32.2% risk of AML evolution, comparable to the 40.4% risk observed for *TP53* multi-hit patients and a shorter mOS [7]. In addition to these *TP53*-mutant patients, patients with *SF3B1* and *RUNX1* mutations also displayed an increased risk of AML evolution [7].

*RUNX1* is mutated in 1–3% of MDS patients with isolated del(5q) and its presence is associated with a reduced response to lenalidomide, shorter OS and increased tendency to AML progression [90,93,95]. Patients who became resistant to lenalidomide harbor recurrent *TP53* (53%) or *RUNX1* (13%) mutations [105]. Experimental studies showed that lenalidomide upregulates RUNX1 protein function in a CRBN- and TP53-dependent manner in del(5q) cells, and the mutation or downregulation of *RUNX1* rendered the cells resistant to lenalidomide [105]. It was shown that cell-intrinsic innate immune signaling driven by *miR-146a* deletion, an event commonly occurring in MDS-del(5q), cooperates with mutant *RUNX1* to initially induce marrow failure and MDS-like condition, progressing with time to AML [106].

About 25–30% of MDS-del(5q) patients are refractory/resistant/ineligible to lenalidomide and will remain dependent on RBC transfusions; for these patients, alternative treatment options are required.

One possible treatment could involve Luspatercept, a recombinant fusion protein that binds endogenous TGFβ superfamily ligands and promotes both early- and late-stage erythroid maturation. The COMMANDS phase II trial showed that Luspatercept achieved significantly greater rates of RBC transfusion independence versus Epoietin alpha, and these effects in responding patients were maintained at long-term [107,108,109]. However, this trial, as well as trials based on Luspatercept administration, enrolled low-risk MDS patients, excluding MDS-del(5q) patients [110]. A single-arm, multicenter study is evaluating the efficacy of Luspatercept in reducing RBC transfusion dependency in MDS patients with del(5q) refractory/resistant/intolerant to prior treatments [111]. Preliminary results on this trial showed a positive signal of efficacy and safety [111]. Patsialos et al., in a case report study, showed the remarkable response to the treatment with Luspatercept in an MDS-del(5q) patient who was refractory to treatment with ESAs and Lenalidomide [112]. The patient achieved a remarkable erythroid response to Luspatercept after only five cycles of treatment [112]. Remarkably, the patients showed a trilineage response with normal hemoglobin levels and increased platelet and neutrophil counts, with no signs in the bone marrow of dyserythropoiesis, normally maturing megakaryocytes and granulocytes and <1% blasts, with a normal karyotype [112]. This response was maintained even fourteen months after Luspatercept discontinuation [112].

The treatment of MDS patients with del(5q) associated with CK alone or in association with *TP53* alterations is highly challenging. The standard of care for these patients, as well as for other high-risk MDS patients, is based on monotherapy with a hypomethylating agent (either azacitidine or decitabine). The AZA-001 phase III study showed that azacitidine prolonged the survival of high-risk MDS patients ineligible for HSCT to 24.5 months compared with 15 months observed for patients treated with low-intensity conventional chemotherapy and supportive care regimens [113]. However, other studies have shown lower overall survival levels compared to those observed in the AZA-001 study [114,115].

A more recent study explored the safety and efficacy of adding the BCL2 inhibitor Venetoclax to the hypomethylating agent Azacitidine. A single-arm phase Ib study showed in 107 high-risk MDS patients treated with Venetoclax + Azacitidine a CR rate of 29.9% and CR with incomplete count recovery (CRi) of 48.6%; the mOS at 26 months was good, and 39% of patients went on to receive HSCT [116]. In the phase III VERONA trial, the patients with high-risk MDS were randomized to Azacitidine and Venetoclax or Azacitidine alone; however, the results of this study, recently presented at the Society of Hematologic Oncology (SOHO) Annual Meeting (Abstract MDS-1497), showed no benefit with the addition of Venetoclax to Azacitidine compared to Azacitidine alone in high-risk MDS. The CRR was superior with Azacitidine plus Venetoclax.

The resistance to Venetoclax of MDS patients with biallelic *TP53* alterations is in part related to the high expression of BCL-X_L_, an anti-apoptotic protein [117]. BCL-X_L_ hyperexpression in these MDS cases is associated with an erythroid phenotype [117]. This observation suggests the potential use of BCL-X_L_ inhibitors in the treatment of these patients.

As discussed above, the group of MDS associated with CK represents a heterogeneous entity subdivided according to the presence of *TP53* mutations: the group CK-*TP53*-mutant had more frequent del(5q) and a significantly shorter mOS than the group CK-*TP53*-WT [9,10]. Considering these differences, Huber et al., in their MDS classification, have proposed two distinct groups: one bearing *TP53* biallelic alteration and the other bearing complex karyotype without *TP53* alterations [118]. Both these MDS groups have a poor prognosis, but the mOS of biallelic *TP53* MDS is shorter than that of MDS with CK [118]. As reported above, the molecular taxonomic study hierarchically classified MDS into molecular taxonomic groups according to mutational clusters; one of these clusters is multi-hit *TP53* or CK [5]. Molina and colleagues evaluated the response to HMAs of a group of 268 patients classified according to the molecular taxonomy; the *TP53*-multi-hit or CK and *EZH2-ASXL1* groups exhibited the poorest OS (1.26 and 0.84 years compared to 4.74 years of the whole MDS cohort) and the highest risk of AML transformation [119]. A recent study evaluated the prognostic impact of the molecular taxonomy classification in a group of 484 patients and observed that the *TP53*-multi-hit or CK group is heterogeneous in that *TP53*-mutated patients exhibited a significantly shorter OS than those with CK without *TP53* mutations [120]. Importantly, in the *TP53*-multi-hit or CK group, the survival probability of these patients was not affected by blast number, either <5% or >5% [120]. Huber and colleagues reported blast cell numbers for various molecular subgroups of MDS patients and observed that blast cell number is highly variable in both the CK and biallelic *TP53* groups, with a mean blast number higher for biallelic *TP53* than for *CK* MDS [119]. The blast cell number could help to identify the stage of disease within these two MDS groups [121].

The relevance of blasts quantification in the classification of MDS is questionable. Thus, while it is evident that the relevance of blast cell quantification as a prognostic factor in the context of different MDS cases, the significance of blast counts is variable for different MDS genetic groups [122].

The group of MDS patients with CK is heterogeneous, and a recent study reported the identification of a subset of these MDSs, characterized by absent *TP53* mutations or *TP53* deletions due to chromosome 17 loss but exhibiting a dysfunction of p53 protein [123]. Thus, in a recent study, Zampini et al. reported the molecular characterization of 6204 MDS patients with particular emphasis on the characterization of MDS exhibiting dysfunction of p53 related to genetic alterations or to altered expression/function [123]. In this study, the exploration of 42 MDS patients with *TP53* mutations and displaying AML evolution showed the following: 23 patients exhibited monoallelic *TP53* alterations at diagnosis, 18 progressed to a biallelic status at AML evolution; and 19 patients displaying AML progression showed an increased *TP53* mutation VAF and acquisition of additional chromosomal abnormalities [123]. These observations strongly support the view that mono-allelic and bi-allelic *TP53* alterations represent different stages occurring through a process involving multiple hits during the natural evolution of MDS. These authors identified a subgroup of MDS characterized by *TP53*-WT and hyperexpression of p53 protein in bone marrow, constantly associated with CK; chromosome 5 abnormalities were observed in about half of these patients; *TET2* (11%), *ASXL1* (11.1%) and *RUNX1* (12.2%) were the genes most frequently mutated [123]. Patients with *TP53*-WT and p53 protein hyperexpression displayed a dismal outcome, comparable to that observed in patients with biallelic *TP53* alterations [123]. The increased p53 protein expression observed in these MDS was associated with absent activation of p53 targets, thus suggesting that p53 protein, although non-mutated and hyperrepressed, is abnormal and not functional [123]. These patients displayed several p53 upstream alterations at the level of PI3K cascade, RAS, WNT and NF-kB pathways, as well as MDM2 gene amplification [123]. Furthermore, MDS with p53 hyperexpression and *TP53*-WT exhibited a peculiar immune dysregulation involving myeloid-derived inflammation and impaired antigen presentation [123].

Few studies have specifically explored the outcome of MDS with CK. Rasmussen et al. have reported the results of a randomized phase II study involving treatment with Azacitidine alone or Azacitidine + Lenalidomide, involving the enrollment of 72 MDS or AM (75% MDS) patients with a karyotype including del(5q) (83% with CK, 76% with *TP53* mutations in 96% of multi-hit cases) [124]. The ORR in the treated cohorts was 39% for AZA and 44% for AZA + LEN, with a CRR of 17% and 28%, respectively [124]. The mOS was 115 months for the whole population and 13.6 months in the AZA arm and 10.8 months in the AZA + LEN arm [124]. In a subsequent study, the same authors have explored the response of these patients according to their karyotype, defined by standard karyotyping analysis and FISH: the ORR did not differ between patients with <3 aberrations and patients with >3 aberrations; patients with >3 aberrations displayed shorter overall survival (9.9 months) compared to those with <3 aberrations (25.2 months) [125]. Patients with unbalanced translocations of 5q have significantly shorter OS than those with del(5q) (8.4 months vs. 21.1 months, respectively) [125]. Unbalanced 5q translocations were more frequently associated with CK and multi-hit *TP53* than del(5q) (for CK, 98% vs. 67%; for multi-hit *TP53*, 88% vs. 47%) [125]. Cytogenetic progression occurred at a similar frequency in patients treated with AZA or AZA + LEN [125]. Thus, according to these observations and other data present in the literature, the CK-MDS can be subdivided into four subgroups: a subgroup with biallelic *TP53* alterations and frequent del(5q), a subgroup with monoallelic *TP53* alterations and frequent del(5q); a subgroup with p53 hyperexpression without *TP53* mutations or chromosome 17 aberrations and with less frequent del(5q); and a subgroup without *TP53* mutations but with frequent chromosome 17 deletions and less frequent del(5q).

## 9. Conclusions

The studies carried out in the last three decades have dramatically contributed to define the molecular spectrum of genetic abnormalities observed in MDSs associated with the presence of del(5q), with the identification of a MDS subtype associated with isolated del(5q) and of a heterogeneous group of MDS in which del(5q) is associated with *TP53* alterations and/or with CK. It is fundamental to distinguish these two types of MDS with del(5q) for their different prognosis and treatments.

The molecular studies performed in patients with isolated del(5q) have contributed to defining the functional role of the various genes present in the deleted regions of 5q, whose loss contributes to a pathogenetic mechanism of contiguous gene effect in which the final hematologic phenotype is dependent upon the collective effect of different gene deletions. However, although del(5q) is a key pathogenic event in 5q^−^ syndrome, some co-occurring somatic mutations, including *TP53*, *SF3B1*, *CSNK1A1* and *RUNX1* mutations, affect the outcomes of these patients, reducing the response to treatment and/or increasing the risk of AML development. Given this heterogeneity of MDS with isolated del/5q, an integration of morphologic, clinical, cytogenetic and genomic data for each patient is required to identify different clinical entities of patients and to monitor their response to treatment.

The other group of MDS with del(5q) associated with CK and *TP53* alterations can be subdivided into four different subgroups according to the *TP53* mutational status: a subgroup with biallelic *TP53* alterations and frequent del(5q) (about 70%, 65% in association with CK and 5% isolated); a subgroup with monoallelic *TP53* alterations and frequent del(5q) (about 50%, 35% in association with CK and 15% isolated); a subgroup with p53 hyperexpression without *TP53* mutations or chromosome 17 aberrations and with less frequent del(5q) (about 35%, always in association with CK); and a subgroup without *TP53* mutations but with frequent chromosome 17 deletions and less frequent del(5q) (<10%, mostly as isolated del(5q)).

Very recent studies support the need to define molecular subgroups of MDS patients according to del(5q) status, other chromosomal abnormalities, *TP53* mutational status and other somatic mutations. Thus, Montoro et al. explored the group of MDS with multihit *TP53* alterations with emphasis on the comparison of the prognosis of the subgroup of patients with *TP53* biallelic alterations and isolated del(5q) and of the subgroup of patients with biallelic *TP53* alterations without isolated del(5q), with low blast cell number [126]. This analysis showed that del(5q) *TP53*^multihit^ MDSs compared to non-del(5q) *TP53*^multihit^ MDSs display the following: (i) an improved overall survival (57.0 months vs. 14.0 months, respectively); (ii) a comparable risk of AML progression at 48 months (34.9% vs. 33%, respectively); and (iii) a markedly longer time of AML progression (31.7 months vs 7.2 months) [126]. These observations strongly support the view that MDSs with isolated del(5q) and with *TP53*^multihit^ represent a clinically distinct entity with better outcomes, and their classification should be carefully reconsidered [126]. Another study explored the group of MDS with complex karyotype, biallelic *TP53* alterations and del(5q) [127]. The group with biallelic *TP53* includes the following: (i) 17p deletion plus *TP53* mutation; (ii) *TP53* mutations with 17 cnLOH; (iii) homozygous *TP53* mutations; and (iv) compound heterozygous *TP53* mutations with two different mutations in trans [127]. A total of 72% of cases with CK had del(5q); *TP53* biallelic alterations were in 85% of CK cases with del(5q); and del(17p) was present in 51% of CK-del(5q) cases [127]. These observations support that biallelic *TP53* aberrations and double *TP53* mutations are prevalent in MDS patients with del(5q)-complex karyotype [127]. These results support the hypothesis that 5q loss cooperates with *TP53* mutations to drive evolution to complex karyotype, as suggested also by experimental studies in induced pluripotent stem cells [128].

The identification of these patients requires an accurate cytogenetic and molecular analysis. The prognosis of these patients is poor, particularly of those with biallelic *TP53* alterations or with p53 hyperexpression. The treatment of these patients is challenging and is mainly based on hypomethylating agents, followed when possible, by allogeneic SCT.

## Figures and Tables

**Table 1:** Some functionally relevant genes, located in the distal and proximal CDRs deleted in MDS-del(5q), involved in the pathogenesis of this myelodysplasia.

Gene	Location	Biological Activity	Gene Knockout Hematologic Phenotype
CDC25C	Proximal CDR (5q31.2)	It regulates the transition from G_2_ to the M phase of the cell cycle	CDC25 knockout mice are viable and display co-alterations of cell cycle; CDC25 haploinsufficiency confers sensitivity to lenalidomide
CTNNA1	Proximal CDR (5q31.2)	Catenin 1 alpha mediates the anchorage of actin filaments and signal transduction	Growth advantage to HSCs
DIAPH1	Proximal CDR (5q31.3)	Cytoskeleton formation Tumor suppressor	Development of age-dependent myelo- proliferation or MDS
EGR1	Proximal CDR (5q31.2)	Transcription factor	Fitness advantage to HSCs
HSPA9	Proximal CDR (5q31.2)	Control of cell proliferation and response to stress, as well as inhibition of apoptosis	Apoptosis of hematopoietic progenitors; block of erythroid maturation
TIFAB	Proximal CDR (5q31.1)	Inhibition of NF-kB signaling	Deregulation of TRAF6, NF-kB activation in HSCs, induction of ineffective hematopoiesis
CSNK1A1	Distal CDR (5q32)	Serine/threonine kinase involved in multiple cellular processes and pathways	CSNK1A1 haploinsufficiency confers growth advantage to HSCs/HPCs
miR-145	Distal CDR (5q33.1)	It targets various tumor-specific genes	miR-145 and miR-146a loss induces dysmegakaryopoiesis, thrombocytosis and innate immune signaling
miR-146a	Distal CDR (5q33.3)	It targets genes involved in regulation of inflammation and innate immune system	miR-145 and miR-146a loss induces dysmegakaryopoiesis, thrombocytosis and innate immune signaling
RPS14	Distal CDR (5q33)	40S ribosomal protein	Macrocytic anemia
SPARC (Osteonectin)	Distal CDR (5q32)	Glycoprotein that binds calcium	Thrombocytopenia Anemia (reduced erythroid progenitors)

**Table 2:** Co-mutations affecting outcomes of MDS-del(5q).

Commutation	Frequency in MDS-del(5q)	Biologic and Clinical Implications
*SF3B1*	15–20%	Concomitant *SF3B1* mutations are associated with a lower response rate to lenalidomide, lower OS and increased risk of leukemic transformation. MDS-del(5q)/*SF3B1*-mutant cases are frequently associated with *TP53* and *RUNX1* mutations and display phenotypic properties of both *SF3B1*-mutant and MDS-del(5q).
*TP53* (monoallelic mutation)	15–20%	Clinical impact of concomitant *TP53* mutations depending on VAF of mutant allele: <20% no effect on AML transformation rate and OS; >20% increased AML transformation rate and shorter OS. MDS-del(5q) with concomitant *TP53* mutations has a trend toward a reduced rate of response to lenalidomide.
*RUNX1*	1–3%	*RUNX1* mutations are associated with reduced response to lenalidomide, reduced overall survival and a high risk of AML progression.
*CSNK1A1*	8–10%	*CSNK1A1* mutation occurring at the level of the non-deleted *CSNK1A1* allele is associated with reduced response to lenalidomide and increased risk of progression.

## Data Availability

No new data were created or analyzed in this study. Data sharing is not applicable to this article.
